# Impact of deep ocean mixing on the climatic mean state in the Southern Ocean

**DOI:** 10.1038/s41598-018-32768-6

**Published:** 2018-09-27

**Authors:** Hiroaki Tatebe, Yuki Tanaka, Yoshiki Komuro, Hiroyasu Hasumi

**Affiliations:** 1 Japan Agency for Marine-Earth Science and Technology, Yokohama, Japan; 2 Graduate School of Science, University of Tokyo, Tokyo, Japan; 3 Atmosphere and Ocean Research Institute, University of Tokyo, Kashiwa, Japan

## Abstract

The Southern Ocean is of great importance for the global stratification and biological carbon storage because it is connected to the global ocean conveyor by which atmospheric information absorbed in the Southern Ocean is redistributed globally and buffered over centuries. Therefore, understanding what controls the Southern Ocean climate, the global ocean conveyor, and links between them is a key to quantifying uncertainties in future climate projections. Based on a set of climate model experiments, here we show that the tide-induced micro-scale mixing in the Pacific deep ocean has significant impacts on the wintertime Southern Ocean climate through basin-scale reorganization of ocean stratification and resultant response of the global ocean conveyor. Specifically, Pacific deep water, which is modified by the deep ocean mixing while travelling south, reinforces the subsurface stratification and suppresses deep convection in the Southern Ocean. Resultant increase of the Ross Sea sea-ice leads to decrease of incoming shortwave radiation and strengthening of the westerly and storms. Because the Southern Ocean could regulate the global warming progress through its role as heat and carbon sink, our study implies that better representation of deep ocean mixing in climate models contributes to reliability improvement in regional-to-global climate projections.

## Introduction

The Southern Ocean dominates the global ocean heat and carbon uptake through wintertime mixed layer deepening and the resultant subduction of surface waters to the thermocline and intermediate layers^1–3^. Atmospheric heat and carbon taken into the Southern Ocean are redistributed to all of the world’s oceans by the global meridional overturning circulations (MOC), namely, the global ocean conveyor^4,5^, which controls the global ocean stratification and biological carbon storage^2,6–9^. For these reasons, the Southern Ocean is of great importance in determining Earth’s climatic mean state as well as climate responses to increasing anthropogenic greenhouse gas emissions^10–13^.

As has been widely recognized, there are several common problems which are encountered when modeling the Southern Ocean. For example, poor representations of mixed layer depths and open ocean deep convection are well-known issues in global ocean modeling, and they are due to lack of mesoscale processes in the Antarctic Circumpolar Current^14,15^, uncertainties in surface fluxes^16^, and other missing physics in the ocean. Another issue is overestimation of incoming solar radiation (ISR) mainly due to cloud radiative processes in atmospheric modeling^17,18^. These systematic errors commonly found in climate models cause a warm sea surface temperature (SST) bias in the Southern Ocean and associated underestimation of sea-ice area. Sea-ice acts as an insulator at the sea surface and, together with wind-speed, air-sea temperature differences, and carbon concentration^16^, is one of the controlling factors which determine air-sea heat and carbon exchange amounts. Therefore, the underestimation of the sea-ice area in the Southern Ocean constitutes serious obstacles to reducing errors and uncertainties in global warming projections.

The oceanic hydrographic structure and sea-ice distribution in the Southern Ocean are influenced by various water masses of the global ocean. Deep waters of polar ocean origin gain buoyancy in the interior ocean by micro-scale vertical mixing of sea water due to the breaking of tide-induced internal waves around rough bottom bathymetry^19–21^. In the Pacific, this tide-induced mixing causes buoyancy-forced basin-wide upwelling of Circumpolar Deep Water (CDW)^22,23^. This upwelling and associated southward flow directed to the Southern Ocean, which compose the Pacific MOC and constitute a part of the global ocean conveyor. The modified CDW then joins the thermocline and intermediate layers along the path of the Pacific MOC. After reaching the Southern Ocean, it is brought to the sea surface by wind-induced upwelling^5^. The upwelled water is then transformed to thermocline and intermediate waters in the Southern Ocean by surface buoyancy fluxes^5,24^. The stratification in the Southern Ocean, which is determined by the above-mentioned processes, is an important controlling factor for heat exchange between sea-ice and subsurface water through isolating the relatively cold surface water from the warm deep water^25^. Therefore, the strength of the stratification can significantly influence SST and ISR in the Southern Ocean.

In the present study, focusing on the Pacific part of the global ocean conveyor, possible impacts of the deep ocean mixing on the wintertime sea-ice area of the Southern Ocean and the resultant atmospheric responses are explored based on a set of climate model experiments. A special emphasis is laid on the representation of deep ocean mixing in climate models, which can be a crucial factor for quantifying uncertainty in the driving processes of the Southern Ocean climate.

## Results

### Sea-ice and ISR in the southern ocean

Climate model experiments are conducted with two different representations for the tide-induced deep ocean mixing. The first is called the TED experiment, where a global map for the tidal energy dissipation rate (Fig. S1) obtained from a global three-dimensional tide model^26,27^ is employed when estimating eddy vertical diffusivity, *K*_*v*_. The other is called the CTRL experiment, where a vertical one-dimensional empirical profile is prescribed for *K*_*v*_. This empirical profile was introduced to obtain the observed strength of the Pacific MOC while taking account of the observed bottom-intensified mixing features^28^. Experimental setting details are described in the Methods section.

**Figure 1:**
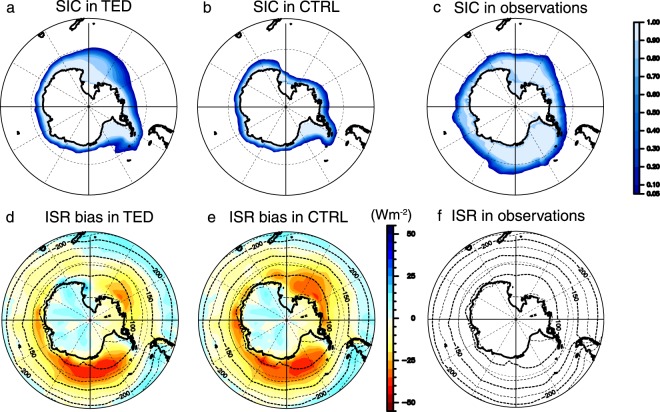
Impact of deep ocean mixing on the Southern Ocean climate. (**a–c**) Wintertime sea-ice concentrations (SIC). (**d**,**e**) Annual-mean ISR bias (shades; upward positive) with respect to observations shown in (**f**). Climatological ISR is indicated by contours. This figure was prepared with GFD-Dennou Common Library version 7.1 (Free Software - http://www.gfd-dennou.org/library/dcl/LICENSE).

As shown in Fig. 1, there are significant differences between TED and CTRL in the Southern Ocean climate. The wintertime (July-September) sea-ice area in the Ross Sea for TED extends further northward than in CTRL. This increased sea-ice area leads also to an increase of the surface albedo, which results in an ISR decrease. The annual-mean ISR bias with respect to Earth Radiation Budget Experiments (ERBE^29^) is 10.6 Wm^−2^ downward in TED and 14.3 Wm^−2^ downward in CTRL over the Southern Ocean to the south of 50°S. This indicates a 30% bias reduction in TED.

**Figure 2:**
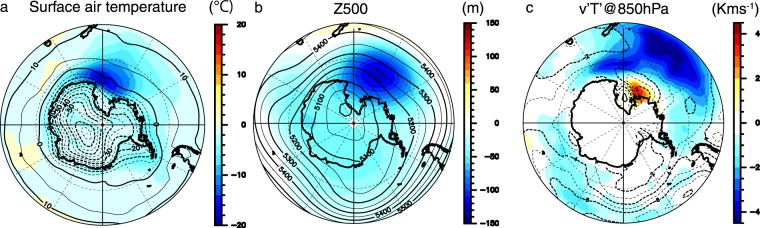
Indirect influence of deep ocean mixing on atmospheric circulations over the Southern Ocean. (**a**) Wintertime difference of the surface air temperature (shades) between TED and CTRL. (**b**,**c**) Same as in (**a**), but for the 500 hPa height and storm track activity. Storm track activity is measured as the meridional eddy heat flux at the 850 hPa level. To extract atmospheric transient eddies, a high-pass-filter with a cut-off period of 8 days is applied to daily-mean data. In (**a**–**c**) values in CTRL are denoted by contours. This figure was prepared with GFD-Dennou Common Library version 7.1 (Free Software - http://www.gfd-dennou.org/library/dcl/LICENSE).

The larger sea-ice area in TED results in the lower surface air temperatures over the Ross Sea (Fig. 2a) because the sea-ice cap reduces turbulent heat fluxes from the ocean to the atmosphere. The area-mean wintertime surface air temperature to the south of 50°S is 2.9 K colder in TED than in CTRL. This temperature difference reaches the middle troposphere. The wintertime westerly jet and the activity of the sub-weekly storms over the Ross Sea are enhanced in TED (Fig. 2b,c). Note that these differences have little impact on the mean climate outside the Southern Ocean. Although the surface air temperature difference between the two experiments extends northward and spreads in the Southern Hemisphere, the precipitation difference outside the Southern Ocean does not correspond to the surface air temperature difference (Fig. S2a,b). In addition, the Pacific zonal-mean precipitation difference apart from the Southern Ocean is within one standard deviation in CTRL and is not significant (Fig. S2c). It is probable that a difference in the ISR between the two experiments is compensated by that in a meridional ocean heat transport, not by atmospheric heat and moisture transports. Correspondingly, the atmosphere outside the Southern Ocean might be insensitive to the difference in the climatic mean state in the Southern Ocean. Because detailed investigation of oceanic compensation mechanisms is reported in previous modeling studies^30,31^, we will not discuss the issue here.

Systematic errors in cloud radiative processes in climate modeling have been considered to be the main candidates for the ISR bias over the Southern Ocean^32,33^. More specifically, underestimation of low- and mid-level clouds associated with the cold fronts of transient cyclones in the mid-latitude westerly jet could be responsible for the ISR bias up to 50% compared with observations^32,33^. Here, we show that deep ocean mixing also has significant impacts on the climatic mean state in the Southern Ocean, along with cloud processes in the atmosphere. In the remainder of this paper, we investigate how the deep ocean mixing can influence the Southern Ocean climate through the modification of the global ocean conveyor.

### Deep ocean mixing and global ocean conveyor

**Figure 3:**
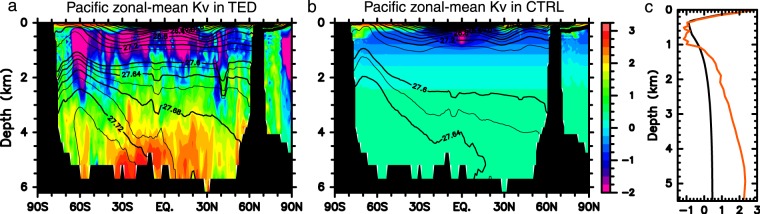
Pacific *K*_v_. (**a**,**b**) Pacific zonal-mean *K*_v_ (shades) and density (contours). The unit is 10^−4^ m^2^ s^−1^ in log_10_. (**c**) Pacific basin average of *K*_v_ for TED (red line) and CTRL (black line). Note that *K*_v_ above the 500 m depth is estimated based on the turbulent closure model of ref.^51^. This figure was prepared with GFD-Dennou Common Library version 7.1 (Free Software - http://www.gfd-dennou.org/library/dcl/LICENSE).

**Figure 4:**
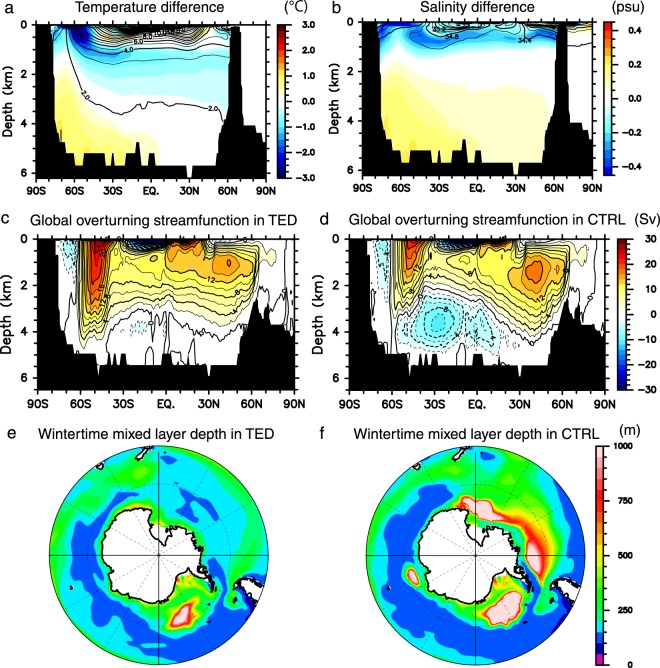
Sensitivity of ocean hydrography, global MOC, deep convection in the Southern Ocean on deep ocean mixing. **(a)** Pacific zonal-mean differences of temperature (TED minus CTRL). Values in CTRL are denoted by contours. (**b**) Same as in (**a**), but for salinity. (**c**,**d**) Global overturning streamfunction. (**e**,**f**) Wintertime mixed layer depth which is defined as the depth where the density is higher than that at the sea surface by 0.125 kg m^−3^. This figure was prepared with GFD-Dennou Common Library version 7.1 (Free Software - http://www.gfd-dennou.org/library/dcl/LICENSE).

Firstly, we summerize the difference in vertical mixing between TED and CTRL. Strong tidal energy dissipation occurs mainly around rough bottom topographies and continental slopes (Fig. S1). Correspondingly, zonal-mean *K*_*v*_ is bottom-intensified in TED and exceeds 10^−2^ m^2^ s^−1^ below the 4000 m depth (Fig. 3a). *K*_*v*_ in TED takes its minimum at the intermediate depths, 500–1000 m and around the density surfaces of 26.8–27.2 *σ*_*θ*_ in the region between 60°S and 60°N, which is clearly seen in the Pacific basin-average of *K*_*v*_ (Fig. 3c). The main reason for this is that the distance between intermediate depths and the sea floor is significantly larger than the vertical decay scale of the internal tidal energy dissipation. Due to the smaller *K*_*v*_, the zonal-mean temperature in the Pacific is warmer (colder) above (below) the 1000 m depth in TED than in CTRL (Fig. 4a). In particular, the deep water warming in TED is restricted below the 4000 m depth to the north of 30°S, consistent with the observed view of the abyssal overturning shaped by bottom-intensified turbulent mixing along the seafloor^34^. The zonal mean salinity is fresher in TED than in CTRL around the 750 m depth due to reduced vertical mixing with relatively saline waters in the surface layers (Fig. 4b).

Figure 4c,d shows the global MOC as an indicator of the global ocean conveyor. The lower cell of the global MOC is weaker in TED than in CTRL, thereby indicating a weakened equatorward intrusion of CDW in the Pacific Sector. The CDW transport across 30°S in the Pacific is 4 Sv (1 Sv ≡ 10^6^ m^3^ s^−1^) in TED and 12 Sv in CTRL, respectively. Any contributions from parameterized eddy bolus velocities are not included in the global MOC. In order to take account of the eddy contributions and to remove spurious circulations, such as the Deacon cell in the Southern Ocean, the global and Pacific MOCs are evaluated on the potential density coordinate (Fig. S3). The CDW intrusions on the potential density coordinate in both experiments are qualitatively consistent with those on the *z* coordinate, and so is its difference between TED and CTRL. The Deacon cell is much weaker on the potential density coordinate than on the *z* coordinate, suggesting that spurious circulations are removed. Therefore, the Pacific MOC is primarily controlled by the buoyancy gain of CDW due to deep vertical mixing of sea water^28^.

The influences of spatially varying and bottom-intensified tide-induced mixing on the Pacific MOC have been also reported in previous studies, many of which described Pacific MOC differences among cases with three-dimensional *K*_*v*_ which is diagnosed from tidal energy dissipation and vertically one-dimensional *K*_*v*_. In general, the basin average of *K*_*v*_ in deeper layers below the intermediate depths is much larger in the former case than in the latter case. However, the Pacific MOC is not necessarily strengthened in the former case because negatively increased vertical gradient of *K*_*v*_ in the intermediate and deep layers inhibits upwelling of CDW, thereby a weaker Pacific MOC^9,28,35,36^. Also in the present study, the Pacific basin average of *K*_*v*_ and its vertical gradient in TED is much larger than in CTRL (Fig. 3c). Correspondingly, the Pacific MOC becomes weaker in TED than in CTRL.

On the other hand, the maximum North Atlantic Deep Water (NADW) transport in TED is 16 Sv, which is as large as in CTRL (18 Sv). From the view point of global MOC closure^5^, the wind-induced upwelling in the Southern Ocean brings deep water to the ocean surface. A large part of the upwelled water is altered to intermediate and thermocline waters, which are then transported to the North Atlantic in the upper branch of the global MOC and feed the Atlantic MOC. Therefore, as reported in previous studies, NADW intensity strengthens as the wind-induced upwelling and northward Ekman transport in the Southern Ocean increase^37–39^. Since the westerly wind is stronger in TED than in CTRL (Fig. 2b), upwelling around 60°S is correspondingly stronger as well, as shown in Fig. 4c,d. However, as mentioned above, the maximum NADW transport in TED is comparable with that in CTRL. As shown in Fig. 4b, the salinity in the surface layer of the Southern Ocean is lower in TED than in CTRL, and this less salty water is exported northward by the wind-induced Ekman processes in TED, resulting in smaller salinity supply to the North Atlantic. This process possibly counteracts the influence of the stronger westerly wind on the NADW transport. For this reason, the NADW transports in the two experiments remain unchanged.

### Deep convection in the southern ocean

While the empirical *K*_*v*_ ensures the observed strength of the Pacific MOC^23,28^, it could also be a cause of the artificial open ocean deep convection in CTRL. Focusing on the Ross Sea, wintertime deep convection is inhibited as seen in the shallower mixed layer depth in TED than in CTRL (Fig. 4e,f). The open ocean deep convection, which is apparent in CTRL, is rarely observed in the real ocean and is considered to be an artifact of numerical models^40,41^.

Since the wintertime deep convection works to mix the relatively cold and fresh water of the surface layer with warm and saline CDW, the weakening of the convection in TED makes the upper layer temperature and salinity in the Pacific sector of the Southern Ocean colder and fresher in TED than in CTRL (Fig. 4a,b). These differences are the most striking in the surface layer above the 200 m depth. The SST is more than 3.0 K colder in TED than in CTRL, and the sea surface salinity is fresher by more than 0.4 psu.

On the other hand, sea water in the layers below the 2000 m depth becomes warmer and more saline in TED. Because the contribution of salinity is dominant over that of temperature in determining the density in high-latitudes, the upper ocean is more strongly stratified in TED than in CTRL. Buoyancy frequency in the subsurface layer around the 150 m depth is 4.0 × 10^−5^ s^−2^ in TED, which is much larger than 1.0 × 10^−5^ s^−2^ in CTRL. The stronger stratification and the resultant weaker deep convection lead to the colder SST in the Pacific sector of the Southern Ocean and thus the larger sea-ice area in TED than in CTRL. This relationship between the sea-ice area and the stratification is detected also in the observed long-term changes in the Ross Sea^25^. In the next subsection, causes for the above-mentioned differences in the Southern Ocean stratification and deep convection are explained in association with the Pacific MOC.

### Influence of Pacific part of global conveyor to the southern ocean climate

Although *K*_*v*_ to the south of 65°S is larger in TED than in CTRL in the entire water column (Fig. 3a,b) because of the remarkable tidal energy dissipation in the coastal region of the Antarctica (Fig. S1), the wintertime deep convection is suppressed in TED due to reinforcement of stratification in the Southern Ocean (Fig. 4). It is expected that the stratification reinforcement is caused not by the local ocean mixing in the Southern Ocean but by the Pacific MOC which brings temperature and salinity influenced by the deep ocean mixing to the Southern Ocean. In order to elucidate whether the differences of the wintertime deep convection, and thus, the climatic mean differences in the Southern Ocean between TED and CTRL can be attributed to the Pacific MOC, two additional experiments, TED-SO and TED-exSO, were carried out. In TED-SO (TED-exSO), the tidal energy dissipation rate used in TED is adopted when estimating *K*_*v*_ to the south (north) of 55°S, and the empirical profile of *K*_*v*_ in CTRL is adopted to the north (south) of 55°S. This latitude is chosen because it is apart from the northern edge of active convective areas in TED around the Antarctic coast.

**Figure 5:**
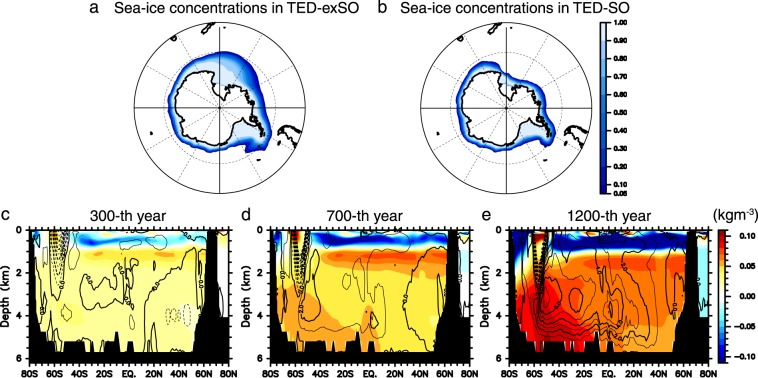
Results of additional experiments and time-evolution of Pacific MOC. **(a**,**b)** Wintertime sea-ice concentrations in additional experiments, TED-exSO and TED-SO. (**c**–**e**) Anomalies of Pacific MOC (contours) and zonal-mean density (shades) in TED with respect to an initial state when TED is branched from CTRL. This figure was prepared with GFD-Dennou Common Library version 7.1 (Free Software - http://www.gfd-dennou.org/library/dcl/LICENSE).

As shown in Fig. 5a,b, the sea-ice area in the Southern Hemisphere in TED-exSO is as large as that in TED after more than 1000 years of integration, but the sea-ice area in TED-SO does not increase and remains nearly the same as that in CTRL. This demonstrates that the Pacific MOC is primarily controlled by buoyancy gain of CDW caused by deep turbulent vertical mixing and influences the climatic mean state in the Southern Ocean. Looking at the time-evolution of the Pacific MOC and zonal-mean density anomalies in TED, density is lowered (raised) above (below) the 1000 m depth and a clockwise anomaly of the Pacific MOC appears first (Fig. 5c). These anomalies continue to develop and the Pacific MOC anomaly works to reduce the northward transport of dense bottom water of the Southern Ocean origin. Accordingly, density in the Southern Hemisphere becomes higher below the 3000 m depth, leading to stratification strengthening, as can be seen in Fig. 5d,e.

Deep ocean mixing brings about significant impacts on the wintertime climate in the Southern Ocean, especially in the Pacific sector. The underestimation of the sea-ice area and the persistent overestimation of ISR in the present climate model can be reduced depending on the representation of the deep ocean mixing.

## Discussion

The Southern Ocean dominates the global ocean heat and carbon uptake; hence, a better understanding for the formation processes of the Southern Ocean climate is essential for reducing uncertainty in transient climate response (TCR) to increasing greenhouse gas emissions^42^, and thus, the global warming projections^43,44^. As mentioned earlier, many of current climate models are suffering from persistent overestimation of ISR and associated underestimation of sea-ice area in the Southern Ocean. In addition, the lack of oceanic mesoscale eddies and errors in surface fluxes in most of climate models cause an unrealistically deep mixed layer distribution and open ocean convections in the Southern Ocean. These systematic climate model errors are obstacles to reducing uncertainty in TCR because they are directly linked to the ocean uptake of heat and carbon^45,46^.

The westerly jet and storm tracks, clouds, and associated radiation budgets over the Southern Ocean are suggested to contribute to uncertainty in TCR^18,47^. Apart from these atmospheric candidates, the present study has demonstrated that the Southern Ocean climate is also sensitive to parameterizations of micro-scale deep ocean mixing. Altering the parameterizations can lead to basin-scale reorganization of the ocean stratification and the resultant Pacific MOC response, resulting in changes in the wintertime sea-ice area, ISR, and the atmospheric circulations. These changes in model climatic mean state imply that ocean uptake of heat and carbon under increasing greenhouse gas emissions could be influenced by deep ocean mixing. In addition to oceanic mesoscale processes and atmospheric responses in a warming climate, micro-scale ocean mixing might be essential to quantifying TCR uncertainties. Furthermore, better parameterizations for unresolved ocean mixing processes could contribute to reducing TCR uncertainties.

## Methods

### Climate model

In the present study, the Model for Interdisciplinary Research on Climate (MIROC) version 5.2, which is a minor upgrade version of MIROC5^48^, is used for the global climate model. The horizontal resolution of the atmospheric component is a T42 spectral truncation (about 300 km), and there are 40 vertical levels up to 3 hPa. The warped bipolar horizontal coordinate system of the MIROC5 oceanic component has been replaced by a tripolar coordinate system. The oceanic component has 1° longitudinal grid spacing in the spherical coordinate portion south of 63°N. The meridional grid spacing varies from about 0.5° near the equator to 1° in the mid-latitudes. There are 63 vertical levels, the lowermost level of which is located at the 6300 m depth.

The ocean component incorporates eddy parameterization for isopycnal tracer diffusion^49^ and isopycnal layer thickness diffusion^50^. The tracer and thickness diffusivities are 10^3^ m^2^ s^−1^ and 3.0 × 10^2^ m^2^ s^−1^, respectively. In addition, the turbulence closure scheme of ref.^51^ is utilized above the 500 m depth to evaluate vertical viscosity and diffusivity in the surface mixed layer. Details for the subgrid-scale parameterizations are described in ref.^48^. In the land surface model, the parameterization for a subgrid-scale snow cover distribution^52,53^ and a simple wetland scheme^54^ have been newly implemented into MIROC5.2. Improved treatment of turbulent kinetic energy input from the atmosphere^55^ are also adopted in the Arctic Ocean sea-ice area.

## Experiments

Breaking of tide-induced internal waves occurring in the scale of a few meters is observed near rough topography^19,21^, and the resultant mixing of sea water is considered to maintain the global meridional overturning circulations^20^. Because the internal wave breaking cannot be resolved explicitly in ocean general circulation models (OGCMs), it has been parameterized as eddy vertical diffusivity, *K*_*v*_. There are several previous approaches for parameterizing *K*_*v*_, by simply prescribing it as a function of depths or bottom roughness^22,56^ or by assuming it to be a function of stability^57^. Recent advances in high-performance computing have enabled estimating global maps for tidal energy conversions from surface to and internal waves by using high-resolution tide models and corresponding maps for *K*_*v*_ have been proposed^27,58^. OGCM experiments which incorporate prescribed global maps for tidal energy conversion have been conducted to address the impacts of sophisticated representation of seawater mixing on the global meridional overturning circulations^9,35,36^.

In the present study using MIROC5.2, we conducted two experiments of CTRL and TED. *K*_*v*_ in TED is parameterized based on a global three-dimensional map for the turbulent energy dissipation rate of the tide-induced internal waves (Fig. S1). The global map is obtained in the same manner described in ref.^26^, but a global three-dimensional model of ref.^27^ is used as a tide model. It is assumed that 30 (100) % of the internal tide energy dissipation for each tidal constituent occurs locally with the vertical decay scale of 500 m from the sea floor if the tidal frequency is super-inertial (sub-inertial) and that the remaining part radiates away to contribute to the background vertical diffusivity of the order of 10^−6^ m^2^ s^−1^. The tidal energy dissipation rate, *ε*, is converted to *K*_*v*_ following ref.^59^ as *K*_*v*_ = 0.2 *ε N*^−2^, where *N* is the buoyancy frequency. *K*_*v*_ above the 500 m depth is replaced by the eddy vertical diffusivity diagnosed using a turbulent closure model if the diagnosed value is larger than *K*_*v*_. In CTRL, *K*_*v*_ is prescribed following an empirical vertical profile proposed in ref.^28^ as

$$0.1+0.9(1+\,\tanh \,\frac{z-1500}{750})\,{\rm{for}}\,z\le 1500,$$

$$-1+2.0(1+\,\tanh \,\frac{z-1500}{2000})\,{\rm{for}}\,z > 1500,$$

where the depth *z* is measured in meters, and the vertical profile is shown in Fig. 3c. This empirical profile considers observed bottom-intensified mixing and ensures realistic reproducibility of the Pacific abyssal circulation. In addition, following the observational fact that the turbulent energy dissipation rate is extremely small in low-latitudes^60^, *K*_*v*_ between 30°S and 30°N is reduced as a function of latitude.

CTRL run is integrated for 2000 years under the pre-industrial forcing at the year 1850, whereas TED run is integrated for 1500 years using the initial conditions of CTRL at the 1200-th year. The last 300-year-long data are analyzed in both of CTRL and TED. The two additional experiments descibed in the text, TED-exSO and TED-SO, are also branched from CTRL and integrated for 1500 years.

## Electronic supplementary material

Supplementary figures
