# Supplementary material for: Impact of deep ocean mixing on the climatic mean state in the Southern Ocean
Source: Sci Rep. 2018 Sep 27;8:14479. doi: 10.1038/s41598-018-32768-6 (PMC6160463; doi:10.1038/s41598-018-32768-6)
Supplement: Supplementary file 1 — Supplementary figures [file 41598_2018_32768_MOESM1_ESM.docx]

Impact of deep ocean mixing on the climatic mean state in the Southern Ocean

Hiroaki Tatebe^1^, Yuki Tanaka^2^, Yoshiki Komuro^1^, and Hiroyasu Hasumi^3^

1: Japan Agency for Marine-Earth Science and Technology, Yokohama, Japan

2: Graduate School of Earth and Planetary Science, University of Tokyo, Tokyo, Japan

3: Atmosphere and Ocean Research Institute, University of Tokyo, Kashiwa, Japan

Corresponding author

Hiroaki Tatebe

Project team for advanced climate modeling,

Japan Agency for Marine-Earth Science and Technology

3173-25 Showa-machi, Kanazawa-ku, Yokohama, Kanagawa 236-0001, Japan

E-mail: tatebe@jamstec.go.jp


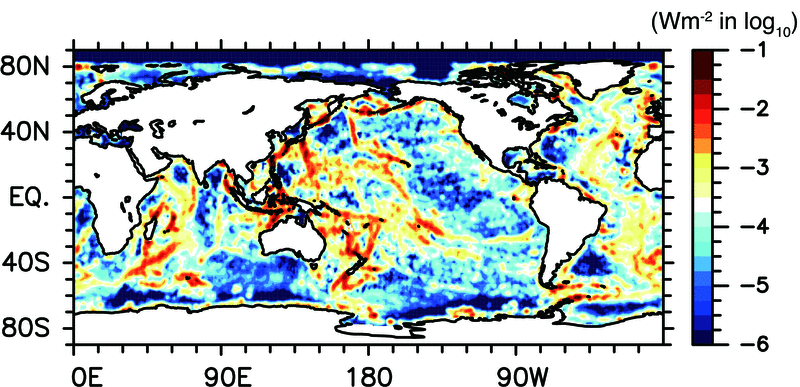


**Supplementary Figure S1. Global map of the depth-integrated tidal energy dissipation rate.** This figure was prepared with GFD-Dennou Common Library version 7.1 (Free Software - http://www.gfd-dennou.org/library/dcl/LICENSE).


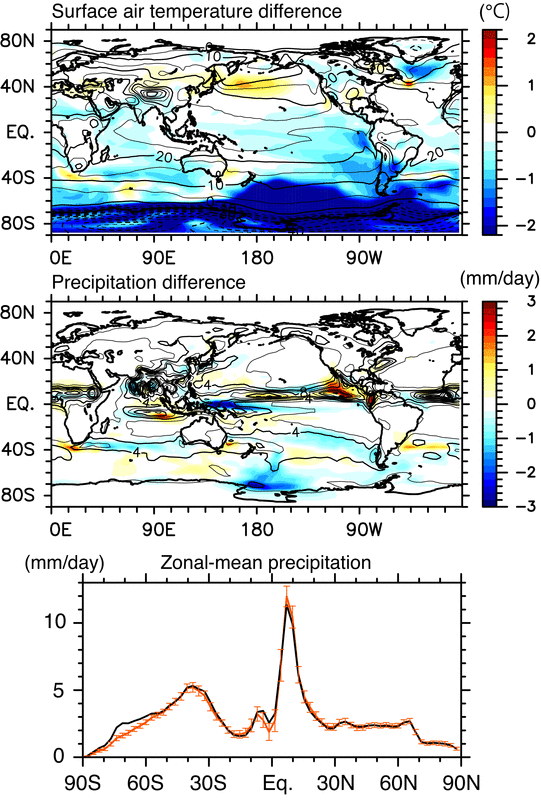


**Fig. S2. Global surface air temperature and precipitation. a,** Wintertime difference of the surface air temperature between TED and CTRL (shades). **b,** Same as **a**, but for precipitation. **c,** Pacific zonal-mean precipitation in TED (red) and CTRL (black). The red bar indicates one standard deviation of the Pacific zonal-mean precipitation in TED. In **a-b**, values in CTRL are denoted by contours. This figure was prepared with GFD-Dennou Common Library version 7.1 (Free Software - http://www.gfd-dennou.org/library/dcl/LICENSE).


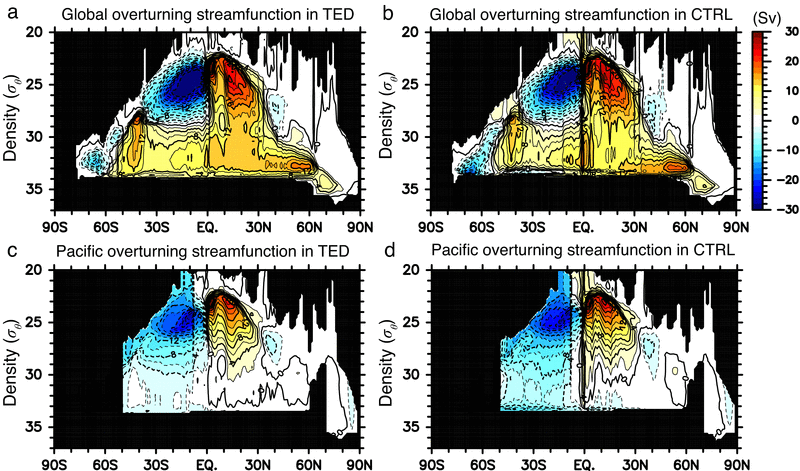


**Fig. S3. Global and Pacific overturning streamfunctions on the potential density coordinate.** This figure was prepared with GFD-Dennou Common Library version 7.1 (Free Software - http://www.gfd-dennou.org/library/dcl/LICENSE).
